# Morphometric Brain Changes in a Merino Sheep (*Ovis aries*) CLN6 Neuronal Ceroid Lipofuscinosis Model

**DOI:** 10.3390/biology15141114

**Published:** 2026-07-10

**Authors:** Amelia Nanni, Emma Elcombe, Maverick Ho Ming Cheung, Timothy Stait-Gardner, Marina Gimeno, Imke Tammen, Marianne D. Keller

**Affiliations:** 1 School of Life and Environmental Sciences, The University of Sydney, Sydney, NSW 2050, Australia; 2 Ingham Institute of Applied Medical Research, Liverpool, NSW 2170, Australia; 3 Nanoscale Organisation and Dynamics Group, School of Science, Western Sydney University, Penrith, NSW 2751, Australia; 4 Sydney School of Veterinary Science, The University of Sydney, Camden, NSW 2570, Australia; 5 Elizabeth Macarthur Agricultural Institute, Department of Primary Industries and Regional Development, Menangle, NSW 2568, Australia; 6 Sydney School of Veterinary Science, The University of Sydney, Camperdown, NSW 2050, Australia

**Keywords:** batten disease, CLN6, cerebellum, arbour vitae, striatum, thalamus, corpus callosum, brain mapping, segmentation, volumetry

## Abstract

The neuronal ceroid lipofuscinoses are the most common group of human paediatric genetic neurodegenerative disorders and have also been reported in multiple animal species. This study explores sheep with the CLN6 disease subtype, which occurs in humans either as a late-infantile or as an adult-onset disease. This study characterised morphometric changes that occur in the brains of 15-month-old Merino sheep with CLN6 disease compared to healthy, adult Merino control brains using ultra-high field magnetic resonance imaging (MRI). The formalin-fixed brains of seven affected and three wild-type control sheep were scanned in a 9.4 T Bruker MRI scanner with a T1-weighted gradient echo. Bioimaging technology ‘Amira-Avizo’ was used to segment each region of interest to create a 3D reconstruction of each ovine brain. Volumetric and statistical analysis of each region of interest found that the thalamus, corpus callosum, occipital cortex, hippocampal region and striatum of affected sheep all experienced a significant loss of volume; 80%, 77%, 73%, 50%, 46% respectively, compared to the control brains. The left side of the affected brain showed a significant reduction of 44%, while the volume of the lateral ventricle non-significantly increased by 43%. However, the cerebellum and arbour vitae of affected sheep lost 15% and 8% of their volume, respectively, which was not significant. These findings are overall consistent with previous gross pathology, histopathology and traditional in vivo MRI findings of ovine CLN6 disease models, but suggest that postmortem ultra-high field MRI of formalin-fixed brains can be a complementary approach to in vivo studies. Volumetric analysis of affected brain regions, including the corpus callosum, thalamus, occipital cortex, striatum, and hippocampal region, can provide valuable biomarkers for assessing the effectiveness of therapeutic interventions in NCL.

## Simple Summary

Neuronal ceroid lipofuscinoses are a group of rare inherited diseases that mainly affect children. These diseases are characterised by a progressive loss of cells in the brain, leading to seizures and increasing loss of movement, vision, and thinking abilities. There is currently no cure, and these diseases lead to the early death of affected children. In this study, we investigated sheep that naturally develop the CLN6 form of this condition. Using very detailed brain scanning and computer-based mapping, affected sheep brains were compared with healthy ones. Several important brain regions involved in movement, memory, and communication became significantly smaller in affected animals, in some cases reduced by up to 80 percent. These findings align with other studies that used different methods to investigate changes to the brain of affected sheep and confirm that the disease leads to widespread shrinkage of key brain areas, such as the thalamus, corpus callosum, occipital cortex, hippocampal region and striatum. These brain regions can provide valuable biomarkers for assessing the effectiveness of therapeutic interventions in future studies. Importantly, our results show that studying formalin-fixed brains with advanced imaging can provide accurate information.

## 1. Introduction

Neuronal ceroid lipofuscinoses (NCL) are a group of inherited lysosomal storage diseases caused by variants in at least 13 different genes that together represent the most common group of human paediatric genetic neurodegenerative disorders, present in approximately 1/100,000 human births [1]. Children with CLN6 disease [2] caused by variants in the *CLN6* gene present with early onset of clinical signs between 18 months and eight years old. Seizures, progressive regression of motor functionality, cognitive decline, visual impairment leading to blindness and speech impairment are the most common clinical signs, as well as limited life expectancy of children with CLN6 disease of three to five years after diagnosis [3]. Some *CLN6* variants cause an adult-onset form of NCL called Kufs Type A disease [2,4], characterised by progressive myoclonus epilepsy, dementia and reduced life span. Currently, there are no curative or effective treatments for any form of NCL, but several experimental therapeutic interventions are being trialled in animal models, and some have progressed to phase I/II clinical trials in human patients [1].

Naturally occurring or genetically engineered forms of NCL have been reported in many animal species [5,6]. Merino and South Hampshire sheep have been found with disease due to two different *CLN6* variants [7,8]. In White Swedish Landrace sheep, the disease is caused by a *CTSD* variant [9], and Borderdale sheep have been identified with CLN5 disease [10]. These sheep are valuable translational models for the corresponding human diseases [11]. In these ovine models, disease has a recessive mode of inheritance, causal variants have been identified, and clinical presentation and pathology have been characterised. Borderdale sheep with CLN5 disease have been used to evaluate the safety and effectiveness of gene therapy [12,13].

In contrast to rodents, sheep represent a larger model size with easily differentiated neuroanatomical structures [14]. Their long-life span facilitates longitudinal studies, and their current availability and similarities in clinical presentation and pathology make sheep the most advantageous model for CLN6 disease [11,15]. Furthermore, sheep are useful for therapeutic efficacy studies, as the dosage mechanisms more accurately reflect those in humans compared to smaller animals such as mice [14,16]. Sheep have an anatomical brain structure similar to humans, making them effective preclinical models to study neuropathological diseases [17]. Their brains exhibit a prominent motor cortex, clearly delineated striatum, corpus callosum, thalamus, substantia nigra and a hippocampus comparable to that of humans [11,18,19,20]. Sheep have similar spine lengths, spinal canal width and cerebrospinal fluid volume compared to humans [21,22]. Overall, this is supported by the homology (>85%) between human and ovine genomes [11]. This conveys that sheep with CLN6 disease could reliably reflect the morphometrics and neuronal pathways that are also affected in human CLN6 disease.

This study investigates the effect of CLN6 disease on the morphometrics of specific brain structures in CLN6-affected Merino sheep using ultra-high field MRI. In MRI studies with three-dimensional (3D) datasets, a region of interest (ROI) is a 3D volume chosen for focused analysis. The corpus callosum, striatum, thalamus, hippocampus and occipital cortex were selected as ROIs for observation in Merino sheep with CLN6 disease, as they could be expected to decrease in volume [22,23]. In ovine studies by Mitchell et al. [24] and Murray et al. [25], the cerebellum and arbour vitae are relatively spared from atrophy in cases of ovine CLN6 disease. This makes them useful as control ROIs that could reflect the extent and progression of atrophy in other affected structures within the brain.

This is the first report of high-resolution morphometric analysis through brain mapping of ex vivo 9.4 T MRI data from formalin-fixed brain tissue in Merino sheep with CLN6 disease. Results were compared to previous histopathological and lower-resolution MRI studies in Merino, South Hampshire and Borderdale sheep with CLN6 and CLN5 disease [24,25,26].

## 2. Materials and Methods

### 2.1. Acquisition of Merino Sheep Brain Tissue

This study utilised existing biobanked formalin-fixed brain tissue from ten Merino sheep that were bred and maintained as the University of Sydney Merino NCL research flock. Animal breeding and procedures were approved by the University of Sydney animal ethics committee (Project numbers: 2020/1714, 2015/827, 2015/808). The ten Merino sheep used in this experiment were born in 2017 and cared for in accordance with the Australian Code of Practice for the Care and Use of Animals for Scientific Purposes, using standard sheep husbandry and management procedures. A direct DNA test was performed to determine the presence of the Merino *CLN6* variant, NM_001040289.1:c.184C>T (omia.variant:234) [7]. Whole brains were available from two male and five female affected Merino sheep, which had been identified as homozygous for the *CLN6* variant. Affected animals were between 14 and 15 months of age—corresponding to a late adolescence to early adult human life stage—at the time of euthanasia in February 2019, exhibiting noticeable clinical signs of disease (e.g., loss of herding instinct, visual deficits, twitching of facial muscles, mild ataxia, occasional short episodes of walking in circles). Whole brains from three female Merino sheep that had tested negative for the *CLN6* variant were used as controls. The healthy homozygous wild-type sheep were 42 months old—corresponding to an adult human life stage—at the time of euthanasia in June 2021. The age difference between affected and control animals is not ideal, but both groups were considered sexually mature at the time of euthanasia. Mitchell et al. [24] demonstrated that there was no significant increase in ovine brain weight of normal sheep from 12 months to two years. In our study, the control sheep were from the same flock as the affected sheep, born at the same time, which meant that they were genetically similar to the affected sheep, and management had been the same for both populations despite the age difference.

Euthanasia of clinically normal sheep was conducted by intravenous injection of sodium pentobarbitone (140 mg/kg). Due to anecdotal reports that human and animal patients with NCL disease may metabolise barbiturates differently, an alternative protocol was performed for the affected sheep. Euthanasia was conducted in affected animals by intravenous injection of 1 mg/kg diazepam and 10 mg/kg ketamine to achieve deep general anaesthesia, followed by immediate exsanguination.

At postmortem, the brains of affected sheep showed gross morphological changes, such as a marked decrease in brain mass and regional atrophy, and weighed between 60 and 76 g with a mean of 69.5 g (SD 5.3 g) compared to an expected weight of approximately 100 g for clinically normal sheep at 15 months of age [24].

### 2.2. MRI Scanning and Processing

The Merino sheep brain specimens were fixed in 10% neutral buffered formalin following postmortem examination. A week prior to MRI scanning, the samples were immersed in approximately 1 L saline solution (sodium chloride 0.9%) to improve image contrast. This was exchanged three times at 48 h intervals.

MRI images were acquired using a 9.4 T Bruker MRI scanner (Bruker, Billerica, MA, USA) at the National Imaging Facility Node at the University of New South Wales. A T1-weighted gradient-echo scan was acquired: echo time/repetition time = 17.5/2500 ms, scan time = 52.5 min, slice thickness = 0.8 mm, matrix = 150 × 120, and voxel size = 0.4 × 0.4 × 0.8 mm^3^.

### 2.3. Selection of Regions of Interest (ROI)

The corpus callosum was selected as an ROI due to its major responsibility in motor, cognitive and sensory pathways [27], as it enables the right and left hemispheres of the brain to communicate through over 300 million densely packed axons, and it is the largest white matter structure in the brain [26].

The thalamus was selected as MRI studies in human patients with NCL disease show thalamic hypointensity, and due to its roles in sensory domains, motor function, cognitive function and regulation of pain, mood and motivation [28].

The occipital cortex was selected as the primary visual processing region of the brain. CLN6 disease in Merino and South Hampshire sheep has been associated with visual impairment, and in South Hampshire sheep, histological changes were detected in the occipital cortex at day 12 [29].

The hippocampus has been seen historically as the seat of emotion, response inhibition, general memory and spatial perception, but also learning [30].

The striatum was selected as an ROI within this study for its vital involvement in connectivity with the rest of the brain, as it is a subregion of the basal ganglia [31].

The cerebellum and arbour vitae were selected to observe a structure that likely would not change based on Mitchell et al. [24], who demonstrated in a longitudinal study that the cerebellar cortical thickness was similar in controls, CLN5 and CLN6-affected sheep over a period of 24 months.

Lastly, the lateral ventricle and cerebral aqueduct were also selected as ROI. The size of the lateral ventricles increases in response to loss of adjacent tissue, a pattern that has been documented in CLN6-affected South Hampshire sheep [26].

### 2.4. Segmentation and Morphometric Analysis

The scanned data were processed using bioimaging and data processing software Amira-avizo, version 6 (Thermo Fisher Scientific, Waltham, MA, USA), available at the National Imaging Facility Node at Western Sydney University (Campbelltown campus). The workflow followed standard procedures for image processing and analysis as outlined in the software handbook [32] and as used by our group previously [33] with minor adaptations.

The cerebellum, arbour vitae, thalamus, striatum, corpus callosum, hippocampus, occipital cortex, lateral ventricle and cerebral aqueduct were the ROIs segmented by Amira-avizo, using the segmentation workbox. The right side had a rostral sample taken for histopathological examination; therefore, the whole brain volume was not measured. Therefore, we selected the left side of the brain for volumetry. The striatum of one affected sheep was excluded from the study due to sampling for histopathological examination of this region.

The Michigan State University Ovine Brain Atlas was used as a template to accurately identify, isolate and segment each ROI [34]. Segmentation of the ROI began in the coronal plane view, as per the ovine brain atlas, and was then refined in sagittal and axial planes. Each slice was manually segmented using the ‘Brush tool’ to highlight each voxel within each ROI. Additionally, the ‘Thresholding tool’ was used to confirm the accuracy of the voxels selected using the ‘Brush tool’; by specifying the minimum and maximum image intensity of the specific ROIs on the ‘Masking range slider’ within the ‘Display control’, all voxels within this range were masked with a blue overlay [32]. This technique assured accurate differentiation of brain structures, ensuring all voxels were correctly identified with the right ROI. The combination of overlaying the ovine brain atlas with the MRI scans in the segmentation workshop and using thresholding techniques enabled discrimination between brain structures. This generated a specific segmentation of each ROI.

The segmented slices were interpolated to create a 3D reconstruction of each ROI. Morphological variations between control and affected brain samples were inspected by visual analysis. Side-by-side comparisons of each brain from different viewpoints allowed a visual perception of the morphological changes between the control and affected groups.

The whole brain volume could not be measured because of a biopsy of the right frontal lobe of the affected sheep; therefore, we measured the volume of the left side of the brain. The segmentation was achieved using Amira’s lasso tool guided by a selection of landmark points on the boundary of the midline. Five to ten slices along the brain were chosen, and landmark points on the boundary were added to the “left side” of the brain ROI. The brain was then oriented in the 3D view so that neighbouring landmarks overlapped as much as possible. The lasso tool was then used in the 3D view to select the left side and add it to the material. This process continued, one or two more times, for successive groups of landmarks. This method proved remarkably accurate, and only minor manual corrections using the pen tool were required on some slices to complete the segmentation.

The volume of each structure was calculated using the ‘Material statistics’ function ‘count’, which determined the total number of voxels segmented in each ROI. The total volume of each ROI was subsequently calculated by multiplying each ‘count’ value by the voxel size of 0.128 mm^3^. This was subsequently converted into cm^3^ by dividing each value by 1000. Using cm^3^ as the unit of measurement for recording brain volumes is widely used, making the results obtained more easily translatable to other studies [10].

The final 3D reconstruction of each ovine brain’s ROI was visualised through ‘Project view’. For display purposes, control brains were ‘Smoothed’. ‘Smoothing’ removes a thin surface layer of voxels to enhance image quality. It is important to note that ‘Smoothing’ has no influence on the final volume outcome recorded in the data. Affected brains showed no visual alterations; however, the degradation of some of the ROI was so severe that ‘Smoothing’ any sections eliminated large proportions of tissue volume in the images. This would have reduced the ability to visualise the limited remaining tissue.

Distortions at the boundary of materials with differing magnetic properties, which can be caused by air bubbles in brain tissue, are called susceptibility artefacts. Susceptibility artefacts were differentiated from atrophy due to CLN6 disease by their morphology; susceptibility artefacts presented as large, circular, definitive black holes across the scans [33,35]. Atrophy was characterised by gradual tissue reduction determined using the ‘Masking range slider’ [32].

### 2.5. Statistical Analysis

Statistical analysis for this experiment was performed using R-4.5.2 [36]. Descriptive values for each analysis group and ROI are provided in Table 1. A two-tailed Mann–Whitney U test of independence was used to assess the significance of the difference between control and CLN6-affected sheep (Table 1), with the alpha value set at 0.05. The box and whisker plots show the sample median, first and third quartiles, and the whiskers show the range exclusive of outliers. The shaded points show the exact volumetric measurements of each sample.

Comparable analyses of the two affected male and five female ROI volumes were considered; however, the sample size was too small for statistical analysis. Lastly, to assess for potential confounding between control and affected sheep, a within sheep adjustment was completed using the least affected structure (cerebellum) as the reference volume. Volumes were adjusted using the formula Adjusted volume = ROI volume/Cerebellum volume (Table 2).

## 3. Results

### 3.1. Segmentation and Morphometric Analysis

**Figure 1:**
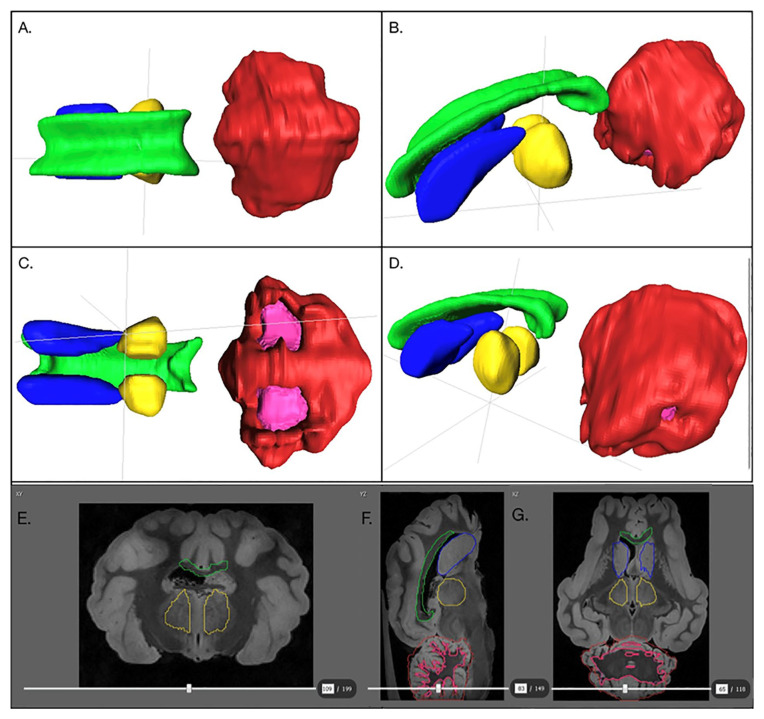
Ultra-high field MRI segmentation of a female control Merino sheep. Normal morphological brain volume and structural architecture were observed in each ROI. ROI colour allocation key: Red = cerebellum, pink = arbour vitae, blue = striatum, yellow = thalamus and green = corpus callosum. Section key: (**A**) = dorsal view, (**B**) = lateral view, (**C**) = ventral view, (**D**) = angled lateral view, (**E**) = coronal plane in MRI view, (**F**) = sagittal plane in MRI view, (**G**) = transverse plane in MRI view.

**Figure 2:**
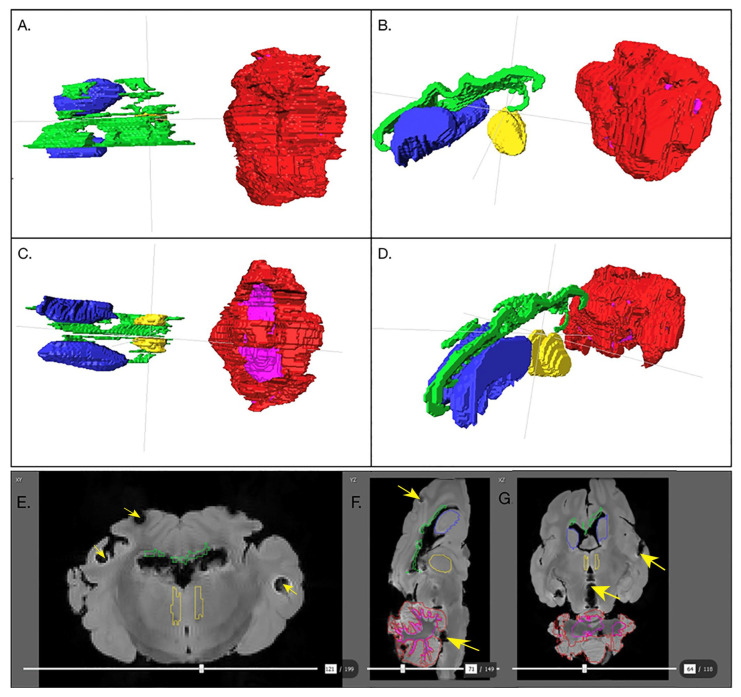
Ultra-high field MRI segmentation of a female CLN6-affected Merino sheep. There is marked volume loss in each ROI. The corpus callosum was the ROI with the highest percentage of volume loss. The normal architecture of the ROIs has been extremely disrupted. Susceptibility artefacts are frequent throughout the cortex and grey matter (yellow arrows on MRI view: (**E**–**G**)). ROI colour allocation key: red = cerebellum, pink = arbour vitae, blue = striatum, yellow = thalamus and green = corpus callosum. Section key: (**A**) = dorsal view, (**B**) = lateral view, (**C**) = ventral view, (**D**) = angled lateral view, (**E**) = coronal plane in MRI view, (**F**) = sagittal plane in MRI view, (**G**) = transverse plane in MRI view.

**Figure 3:**
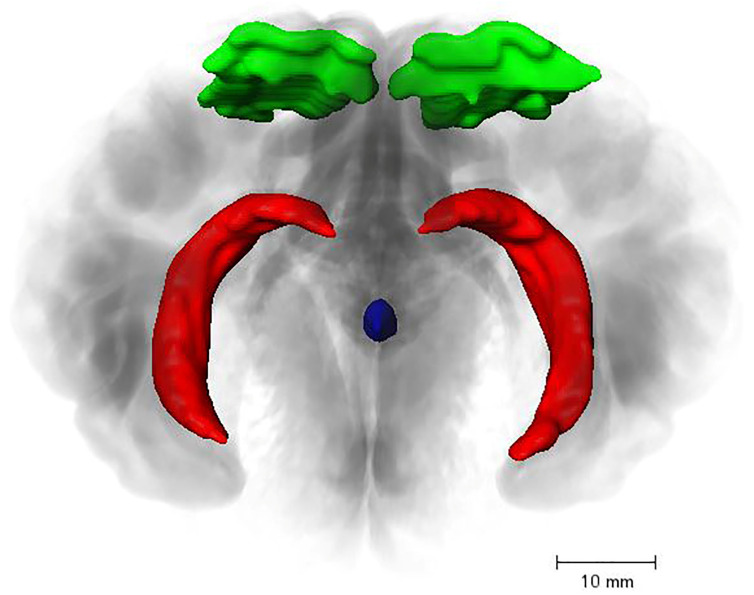
Cranial view of ultra-high field MRI segmentations of a female control Merino sheep. Normal morphological brain volume and structural architecture were observed in each ROI. Overlay of brain tissue in grey on the segmentation of green = occipital cortex, red = hippocampus, blue = cerebral aqueduct.

**Figure 4:**
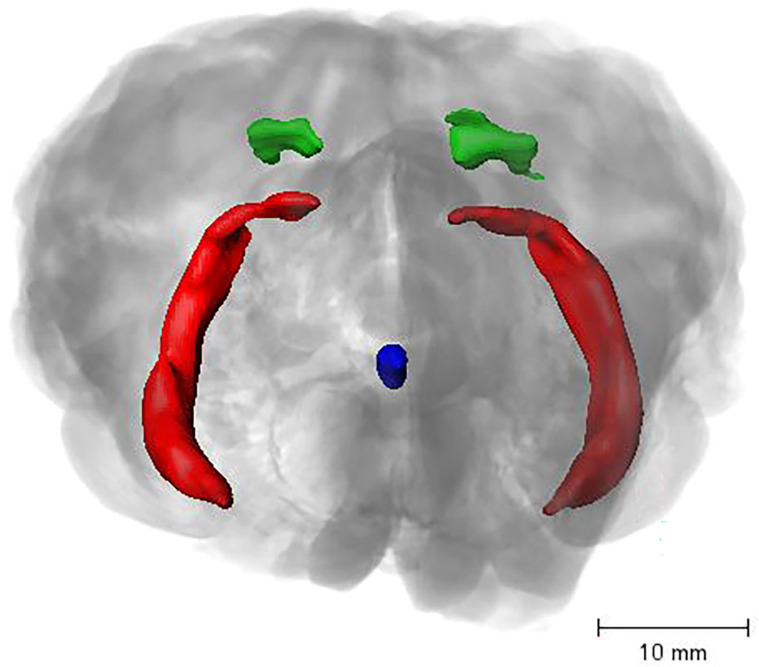
Cranial view of ultra-high field MRI segmentations of a female affected Merino sheep. Reduced morphological brain volume and structural architecture were observed in each ROI. Overlay of brain tissue in grey on the segmentation of green = occipital cortex, red = hippocampus, blue = cerebral aqueduct.

Formalin-fixed brains of three control and seven CLN6-affected Merino sheep were included in this ultra-high field MRI segmentation study. Control sheep presented normal anatomical morphology for each ROI (Figure 1). In comparison, there were marked morphological differences in the corpus callosum, striatum, thalamus (Figure 2), occipital cortex and hippocampus (Figure 3) of CLN6-affected sheep (Figure 4). In CLN6 sheep, the occipital cortex and hippocampus (Figure 4) showed a decrease in volume compared to normal sheep (Figure 3), whereas the cerebral aqueduct did not change in volume or appearance (Table 1). The CLN6-affected group exhibited a substantial decrease in volume and change to the anatomical morphology in the striatum, thalamus and corpus callosum (Figure 2). The corpus callosum showed the greatest volume reduction in all assessed structures, with extensive areas of tissue loss. This was consistent across all CLN6-affected sheep (Figure 2). The two regions that were expected to be least affected by the disease process—the cerebellum and arbour vitae—experienced minor morphometric and anatomical changes across the control and CLN6-affected Merino sheep groups (Figure 1 and Figure 2). Despite the relatively small sample size in this study, statistical analysis suggests that changes to the corpus callosum, thalamus, occipital cortex, striatum, hippocampus and left side are significant (Table 1).

### 3.2. Volumetry Including Statistical Analysis

**Figure 5:**
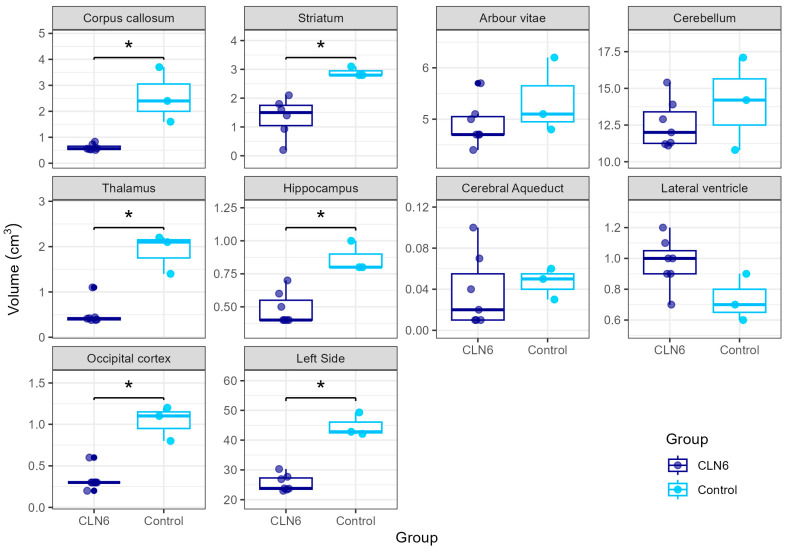
Box and whisker plots showing regions of interest and left side volume measurements for affected CLN6 and control sheep. Shaded points show individual volumetric measurements for each sheep. Whiskers indicate range. * Asterisks indicate statistical significance (*p* < 0.05).

Most ROIs (corpus callosum, thalamus, occipital cortex, striatum, hippocampus) and the left side of the brain in CLN6-affected sheep demonstrated a significant reduction in volume compared to the healthy control sheep (Figure 1, Figure 2, Figure 3, Figure 4 and Figure 5, Table 1).

For the six ROIs most strongly affected by the disease, the volumetric measurements showed perfect separation in the box and whisker plots (Figure 5). This type of clear differentiation is uncommon and a strong indicator of disease impact. In contrast, the data of the arbour vitae and cerebral aqueduct showed significant crossover. The comparatively small 15% reduction in cerebellum volume and the high crossover of volumetric measurement support the previous literature and suggest that the cerebellum is one of the least affected brain structures.

In comparison to control sheep, CLN6-affected sheep showed: a 77% reduction in corpus callosum volume (*p =* 0.017), a 80% reduction in thalamic volume (*p =* 0.022), a 73% reduction in occipital cortex volume (*p =* 0.015), a 50% reduction in hippocampal volume (*p =* 0.018), a 46% reduction in striatal volume (*p =* 0.028), and a 44% reduction in the total left side brain volume (*p =* 0.017).

The observed volumetric changes between control and CLN6-affected sheep that did not reach statistical significance (*p* < 0.05) included a 15% decrease in cerebellar volume (*p =* 0.667), an 8% decrease in arbour vitae volume (*p =* 0.391), and a 60% decrease in cerebral aqueduct volume (*p =* 0.489) (Figure 2, Table 1).

### 3.3. Further Statistical Investigations

**Figure 6:**
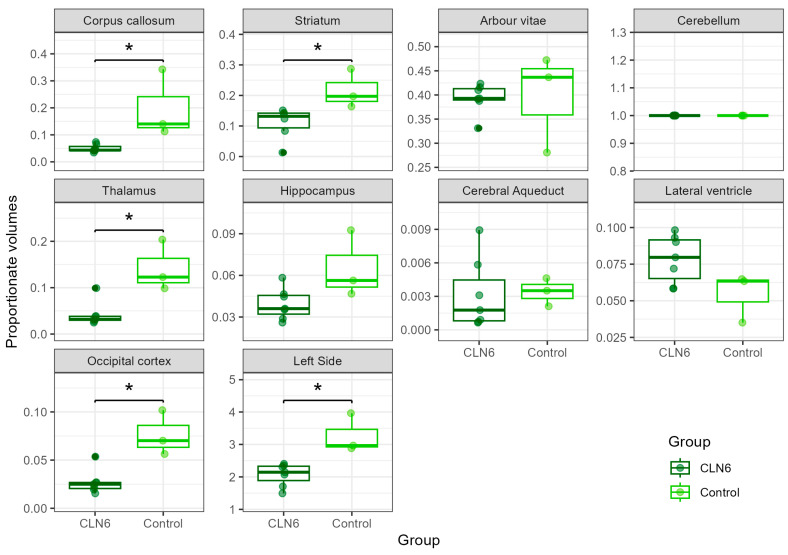
Box and whisker plots showing regions of interest and left side volume measurements for affected CLN6 and control sheep for cerebellum adjusted volumes. Shaded points show individual volumetric measurements for each sheep. Whiskers indicate range. * Asterisks indicate statistical significance (*p* < 0.05).

To assess potential confounding between control and affected sheep, considering the age difference between these two groups, a within sheep adjustment was completed using the relatively unaffected cerebellum volumes (Table 1) as the reference (Table 2, Figure 6). In the adjusted data, CLN6-affected sheep continued to demonstrate a significant reduction in volume compared to the healthy control sheep for five of the six most affected regions: corpus callosum, thalamus, occipital cortex, striatum, and left side of the brain (Table 2, Figure 6). This consistency in significance and the maintenance of almost completely separate score ranges in the box and whisker plots (Figure 6) show that, despite any overall differences in expected brain volume, these regions of the brain are affected. CLN6-affected sheep showed: a 71% reduction in corpus callosum volume (*p =* 0.017), a 75% reduction in thalamic volume (*p =* 0.033), a 71% reduction in occipital cortex volume (*p =* 0.017), a 35% reduction in striatal volume (*p =* 0.024) and a 28% reduction in total left side brain volume (*p =* 0.017).

Post adjustment, the significant difference seen in hippocampal volume disappears, and the ranges start to overlap; this might indicate that hippocampal volume is not as affected by the disease as some other ROIs. Adjustment had little effect on the remaining three regions, with stable, non-significant changes in percentage volume seen. The lateral ventricles of CLN6-affected sheep showed a 33% increase in volume compared to controls (*p =* 0.183), the arbour vitae exhibited an 11% decrease (*p =* 0.517), and the cerebral aqueduct showed a 49% decrease (*p =* 0.517) (Table 2, Figure 6).

## 4. Discussion

### 4.1. Overview

A central innovation of this study was the use of ultra-high field 9.4 T MRI, which, although limited by a bore size unsuitable for in vivo large animal research, provides an improved signal-to-noise ratio. This enables clearer visualisation of fine anatomical details and the detection of subtle morphometric changes that may not be reliably resolved at lower field strengths. Scanners operating at 1.5–3 T are standard for in vivo large animal research; however, safety considerations outweigh the desire to improve signal-to-noise ratio through prolonged scan times aimed at increasing resolution [37,38]. On the other hand, ultra-high field MRI may also enhance tissue contrast in T1-weighted imaging, improving differentiation between soft tissue types with distinct magnetic properties. Generally, ex vivo imaging benefits from a lack of constraints on imaging time, but also the use of tighter fitting coils and a lack of movement artefacts [39].

Post-processing of high-resolution T1-weighted MRI enables quantitative measurement of whole-brain volume as well as detailed morphometric analysis of individual neuroanatomical structures. Previous MRI studies of ovine CLN6 disease focused on longitudinal in vivo studies in South Hampshire sheep using 1.5 T and 3 T MRI, and a preliminary investigation using a 0.25 T MRI in Merino sheep [21,24,25]. This ex vivo experiment aimed to quantify the impact of CLN6 disease on the volume of specific brain structures in Merino sheep using formalin-fixed brains. Previous histopathological and lower-resolution MRI studies suggest these changes could act as biomarkers for disease progression in sheep [23,24,25]. It was expected that control sheep would have normal brain volumes and maintenance of structural integrity, compared to the affected sheep, which would demonstrate significant atrophy, decreased volume and structural loss [23,24,25]. The CLN6-affected Merino sheep exhibited advanced clinical signs of disease. This included loss of herding instinct, visual deficits, twitching facial muscles, ataxia and walking in circles [23,40]. These clinical signs contribute to CLN6-affected sheep consuming less food and moving more, compared to healthy sheep. Mitchell et al. [24] found a reduction in overall body weight, as well as the brain mass in CLN6-affected sheep. Upon postmortem examination, gross morphological changes to the brain were visualised, which aligned with their disease stage progression [24]. Hence, it was expected that severe atrophy would be observed in each ROI.

### 4.2. Thalamus

The thalamus of CLN6-affected sheep showed an 80% volume reduction compared to control sheep (Figure 5). The segmentation of the thalamus showed a significant loss in thalamic volume, but the anatomical morphology and structural integrity were maintained (Figure 2). These results in Table 1 and Figure 2 do not align with a study conducted by Murray et al. [25], which found a non-statistically significant reduction of only 10–13% in the thalamus volume of 18-month-old Borderdale sheep with CLN5 disease and South Hampshire sheep with CLN6 disease. This could demonstrate disparity in the progression of the disease between the breeds with different causal variants. It could also result from the differences in study methods. Murray et al. [25] conducted a longitudinal study on an in vivo tissue MRI study using automated segmentation, compared to the current study using postmortem tissues and manual segmentation. Alternatively, it could be due to the more accurate morphometric analysis in this high-resolution segmentation study.

### 4.3. Corpus Callosum

The corpus callosum showed the highest volume decrease in all ROI, with affected sheep only having 23% of the control volume. The results presented in Table 1 for the 14–15-month-old affected sheep demonstrate significant atrophy and are supported by previous longitudinal ovine studies in CLN5 Borderdale sheep and CLN6 South Hampshire and Merino sheep in which corpus callosum thickness was measured [24]. Furthermore, the segmentation of the affected corpus callosum seen in this study (Figure 2) revealed that the structure and morphology of the corpus callosum were completely compromised. It can be surmised that destruction of neural pathways within the corpus callosum ultimately leads to the degeneration of all connecting white matter pathways across both hemispheres of the brain [27]. This finding aligns with the various neuropathological and clinical symptoms exhibited by CLN6-affected sheep. Mitchell et al. [24] found that the corpus callosum thickness remained relatively unchanged across postnatal development in sheep with CLN6 and CLN5 disease, whereas in normal sheep, thickness gradually increased until 18–24 months old. Corpus callosum thickness in normal 18 to 24-month-old sheep was approximately double compared to that of age-matched affected individuals.

Sawiak et al. [26] also investigated the overall white matter of sheep at around 18 months and found a decrease in white matter between healthy and affected sheep. This suggests that the corpus callosum might not have developed normally, rather than being affected by atrophy. Future studies should consider tracking the white matter pathways from within the corpus callosum to see how other pathways and connecting regions are impacted.

### 4.4. Occipital Cortex

The affected occipital cortex was reduced by 73% (*p =* 0.015) (Figure 3 and Figure 4, Table 1). This correlates with previous studies in sheep with CLN6 disease [24,25,29], which describe the progression of histopathological changes to commence within the parietal and occipital cortex early in the disease process, with glial activation and subsequent atrophy, before spreading to the entire cortex.

### 4.5. Hippocampus and Left Side of the Brain

The affected hippocampal region also showed a volume reduction of 50%, while the affected left side of the brain reduced in overall volume by 44%. In children with CLN3 disease, hippocampal degeneration was found to exceed the loss of overall brain matter in a longitudinal study [41], while in another longitudinal study of human patients with CLN3, a significant decrease in hippocampal volumes was observed [42]. In contrast to our study, Sawiak et al. [26] found no change in hippocampal volume in their in vivo longitudinal study of CLN6 South Hampshire sheep, while noting an overall loss of 40% of cortical volume. This could be due to differences in progression of the disease between the breeds with different causal variants or due to differences in experimental protocols.

### 4.6. Striatum

The striatum experienced a 46% reduction in volume in affected sheep (Figure 1 and Figure 2). Segmentation of the affected striatum also showcased that although atrophy was evident, the general morphology and structural integrity were maintained. There were no compromised regions within the structures; it appeared that the striatum had shrunk and lost volume evenly over the whole structure (Figure 2). These results contradict the outcomes of other studies, which assessed the gradual reduction in basal ganglia volume as a result of the CLN6 disease in South Hampshire sheep. Murray et al. [25] found little to no degeneration of the basal ganglia. Considering the striatum’s connection to the thalamus and other key connective pathways in the brain, as well as being affected by the lowest percentage of volume loss, these results could suggest that the striatum is not a primary structure affected by CLN6 disease progression (Table 1). It is important to note that one CLN6-affected sheep was not included in the striatum volume analysis, as half the striatum had been removed—as part of histological sampling of the brain—which had been completed prior to the commencement of MRI for this study.

### 4.7. Cerebellum

The cerebellum and arbour vitae of Merino sheep with CLN6 disease did not demonstrate a significant loss of volume compared to the control sheep. This aligns with other studies and is consistent across several species; the cerebellum and arbour vitae are spared of significant degeneration in CLN5-affected Borderdale and CLN6-affected South Hampshire sheep, but also in dogs with CLN6 disease [25,43]. However, some variants of human CLN6 present with cerebellar atrophy [44,45]. In affected Merino sheep, there appears to be minor atrophy on the surface of both the cerebellum and arbour vitae, which alters their morphological appearance compared to controls; however, it is unknown whether this minor atrophy contributes to the expression of clinical signs (Figure 2). Previous studies have similarly noted a slight change in appearance; however, the cerebellum is consistently spared from complete regional degeneration [16,46]. This slight change in appearance is reflected in the statistically non-significant decrease in structural volume; 15% for the affected cerebellum and 8% for the affected arbour vitae (Figure 3). The reason why the cerebellum and arbour vitae are less impacted compared to other structures is unknown.

### 4.8. Statistical Evaluations

The relatively small sample size and the age difference between control and affected animals were not optimal for statistical analysis. While we report that results reached statistical significance (*p* < 0.05), these methodological limitations need to be kept in mind.

The sheep in the control group were drawn from the same flock and were born at the same time, ensuring comparable genetics—albeit being homozygous wild-type animals—and both groups were raised under the same conditions. While the age difference between affected and control sheep is not ideal, whole brains from age-matched control sheep with the same genetic background were not available for this retrospective study that used existing biobanked CLN6 brain tissues. Mitchell et al. [24] demonstrated that there was no significant increase in ovine brain mass from healthy sheep from 12 months to two years. However, Mitchell et al. [24] and Cook et al. [46] reported substantial differences in brain mass associated with disease status when comparing control and affected sheep. Data relating to changes to brain weights or volumes in normal sheep, particularly beyond 24 months of age, are difficult to find. Murray and Mitchell [11] state that ovine brain weight increases in normal sheep from less than 1 g in early gestation to approximately 50 g near birth and that adult brain weights of ~130–140 g are reached in adulthood without stating a specific age. Comprehensive longitudinal studies of brain volume, like the human study by Bethlehem et al. [47], which combines ~125,000 MRI scans across the whole human lifespan, are lacking for most animal species. Bethlehem et al. [47] cover brain MRI changes from foetal stages to late adulthood in humans. Rapid and steady brain volumetric increases from foetal age around 17 post-conception weeks, when the human brain is approximately 10% of its maximum size, to the age of 3 years, when the human brain is at approximately 80% of its maximum size, are reported. This is followed by a slower increase for most volumetric brain measurements, which peak around adolescence to young adulthood, followed by a slow decline from mid to late adulthood. Similar studies examining brain-volume changes across the entire lifespan in animals are largely limited to mice [48] and rats [49], while research in dogs has primarily focused on the ageing brain [50]. Across these species, the findings consistently show that global brain volumes reach their peak during adolescence or early adulthood, plateau through the relatively stable “middle-age” period, and then undergo a modest but continuous decline in old age.

While direct age comparisons between species are difficult, we expect that brain volumetric measurements in normal 14–15-month old (late adolescence to early adult) sheep may not differ substantially from 42-month old sheep when considering that sheep have a reported maximum expected lifespan of 22.8 years [51] and are classified as ‘elderly’ when they are older than 7 years [52]. However, in humans, different brain volumetric measurements peak at different life stages [47], and regional remodelling occurs as the brain changes throughout different life stages in mice [48], where ageing-related regional alterations are relatively modest compared with the substantial changes observed during maturation [48].

Therefore, the age difference in affected and control sheep in this study remains a limitation.

To investigate the potential impact of the age difference between affected and control sheep, an additional statistical analysis was conducted. As previously discussed, the cerebellum is spared from significant atrophy in sheep with CLN6 disease [24,25]. We therefore adjusted ROI volumes to the cerebellum volume, creating a ratio (ROI/cerebellum ROI). The analysis of the resulting ratio data showed similar trends (Table 2, Figure 6), indicating that the age difference between control and affected sheep did not have a substantial impact for most ROI, with the exception of the hippocampal volume change, which became borderline non-significant (*p =* 0.067). This result is more aligned with Sawiak et al. [26], who, as discussed above, reported no change in hippocampal volume in their in vivo longitudinal study of CLN6 South Hampshire sheep.

As the age difference between control and affected sheep is acknowledged as a limitation of this study, we cannot demonstrate that the extent of differences in volumetric brain measurements between the two groups is caused solely by CLN6 disease. However, the reported changes correspond to the histopathological abnormalities previously described in affected sheep from the same CLN6 Merino research flock [24,46], and they are consistent with in vivo MRI findings in the CLN6 South Hampshire model [25,26], which is known to exhibit a broadly similar disease progression to the CLN6 Merino model despite being caused by a different *CLN6* variant [24].

### 4.9. Future Directions and Limitations

In vivo MRI preserves native physiological conditions (e.g., blood flow, metabolism, and temperature), enabling longitudinal and functional studies, but is limited by motion, scan time, and lower signal-to-noise ratio (SNR), whereas ex vivo MRI performed on fixed brains allows substantially higher spatial resolution, longer scan durations and scanning at higher field strength, making it particularly valuable for detailed morphometrical analyses [53]. In vivo scan durations are kept short to cause minimal stress to the animal or human patient. Ex vivo imaging better approximates histology in lesion characterisation and reduces confounds such as oedema that can influence in vivo signal contrast [54], while also enabling more reliable quantification of small anatomical structures that are often difficult to resolve in vivo, leading to lower measurement variance compared with in vivo datasets [53].

The impact of tissue processing on volume differences when comparing findings from in vivo and ex vivo studies should be further explored. The literature on fixation-related volumetric changes is variable. Previous studies between in vivo and ex vivo scans showed no change in volumes in murine datasets [55], a reduction in all volumes in a marmoset study [56], whereas there was an increase in ex vivo human hippocampal volume [57]. Because all CLN6 and control brains in the present study underwent the same fixation and imaging protocols, any fixation-related effects would be expected to affect both groups similarly. Therefore, although fixation may influence absolute volumetric measurements and contribute to differences between studies, it is unlikely to explain the regional volumetric differences observed between CLN6 and control sheep.

Future studies could explore whether commonly used fixation processes affect the brain tissue of CLN6-affected and control animals differently, as this may be relevant for studies that aim to quantify disease-related differences. Ma et al. (2019) reported that regions of high cellular density, such as cortical grey matter and subcortical nuclei, may respond differently to fixation than highly myelinated white matter tracts [58]. Cellular membranes in densely packed regions are susceptible to osmotic shifts and protein cross-linking during fixation, potentially leading to greater volumetric contraction, whereas myelin-rich regions may be relatively stabilised by the lipid-rich myelin sheath, which interacts differently with formalin [58], with two studies describing an increase in myelin-rich structures [57,58].

One advantage of higher resolution scans is not only the better visualisation of structures but also a reduction in partial volume artefacts. Partial volume artefacts occur when tissues that have vastly varying density, volume and absorption are collated onto the same voxel so that the average of all these densities is what is shown during processing [37]. Thus, higher resolution scanning may explain why volume changes in some of the ROI in this study (thalamus, hippocampus and striatum) reached significance levels compared to findings in previous lower-resolution scans. The thalamus, hippocampus and striatum showed histopathological changes in CLN6-affected South Hampshire sheep [29]. However, in vivo scans allow for longitudinal studies to monitor the progression of disease and are particularly useful to monitor the efficacy of therapeutic interventions within animals.

A previous in vivo study reported marked widening of the lateral ventricles in CLN5- and CLN6-affected sheep [25]. Although an increase in lateral ventricle volume was observed in affected sheep in the present study (43% in the unadjusted data shown in Table 1 and 33% in the adjusted data in Table 2), these changes were not statistically significant. We hypothesise that reduced cerebrospinal fluid pressure relative to in vivo conditions may have influenced the extent of this increase, suggesting that ex vivo analysis may be less representative for this ROI.

Overall, while both modalities capture consistent large-scale neuroanatomy in large animal brains, in vivo MRI allows for longitudinal studies, while ex vivo MRI provides unparalleled resolution and validation against histology, making the two approaches complementary rather than interchangeable in translational neuroimaging research [54,59].

This study used a T1-weighted gradient-echo sequence, which cannot show the thalamic signal changes reported in human CLN3 disease on T2-weighted scans [41]. Future studies could use a 3D multigradient-echo sequence (with both T2* and T1 weighting) to visualise those changes while still providing the strong T1 contrast needed for detailed brain mapping.

There have been discussions for and against manual segmentation versus automated segmentation through the use of previously published atlases. On one hand, the subjective nature of manual segmentation could increase visual error and individual bias [60]. However, Deeley et al. [61] suggest the need for large datasets and robust statistical analyses to evaluate the quality of atlas-based automatic algorithms. While manual segmentation has been described as the gold standard [62], manual segmentation is time-consuming compared to semi-automated methods [63]. However, in any case, the resolution of the datasets and available anatomical maps must be compatible; otherwise, manual segmentation is required to ensure accurate delineation [39].

The overall brain sample volumes throughout this study may have changed due to formalin fixation [64]. Control sheep were formalin-fixed for a shorter time compared to affected animals. Fixation leads to initial swelling of tissues, followed by shrinkage of brain tissues [65]. However, Del Signore et al. [66] could not find a significant volume reduction in brain regions of in vivo and one month or 12 months post-fixation. Generally, the volume reductions observed in this study align with earlier histological findings in Merino sheep with CLN6 disease [24].

The present study did not aim to address questions of therapeutic windows for human disease, as MRI was conducted at a single time point in affected sheep at 14–15 months of age. Age of onset for human CLN6 patients is reported as ranging between 18 months and eight years, but it most commonly occurs between three and five years of age. Disease progression is rapid, and most children die in childhood between five and 12 years of age [67]. Merino sheep with CLN6 disease show onset of clinical signs at around six months of age, late-stage disease at 18 months of age, and terminal disease at approximately 24 months of age [24,46]. Thus, disease progression in CLN6 Merino sheep at 14–15 months of age could be described as mid-stage disease, corresponding to a CLN6 disease stage seen in early to late childhood in humans. Considering that Borderdale sheep with CLN5 disease and South Hampshire sheep with CLN6 disease have already lysosomal storage body accumulation in neuronal cells, hypertrophic astrocytes and glial activation at birth [24], effective therapeutic interventions in sheep and humans would need to occur as close to birth as possible.

While we recommend considering volumetric measures of the corpus callosum, thalamus, occipital cortex, striatum, and overall brain volume as biomarkers for CLN6 disease studies, and have demonstrated that these ROIs can be effectively assessed in formalin-fixed brain tissue using high-field MRI, future studies with age- and population-matched controls are needed to confirm that the differences reported here are not influenced by age-related changes.

## 5. Conclusions

Ex vivo imaging unlocks the full potential of high-field MRI, enabling longer scan times and dramatically higher resolution than is possible in vivo, where anaesthesia is required, and animal welfare rightly limits duration. This study leveraged those advantages to reveal significant morphometric changes in brain structures affected by CLN6 disease; changes that might remain undetected at lower-resolution in vivo scans.

## Figures and Tables

**Table 1:** Statistical analysis of ultra-high field MRI region of interest (ROI) volumes (cm^3^), analysing seven CLN6-affected Merino sheep and three homozygous wild-type Merino control sheep.

Region	Median Control	Median CLN6	Median Difference	% Reduction	Std. MW-U Statistic ^1^	*p* Value
Corpus callosum	2.4	0.6	1.8	77	2.39	0.017 *
Thalamus	2.1	0.4	1.7	80	2.39	0.022 *
Occipital cortex	1.1	0.3	0.8	73	2.39	0.015 *
Striatum	2.8	1.5	1.3	46	2.34	0.028 *
Hippocampus	0.8	0.4	0.4	50	2.39	0.018 *
Left side	42.8	23.8	19.0	44	2.39	0.017 *
Arbour vitae	5.1	4.7	0.4	8	1.03	0.391
Cerebral aqueduct	0.05	0.02	0.03	60	0.80	0.489
Cerebellum	14.2	12.0	2.2	15	0.57	0.667
Lateral ventricle	0.7	1.0	−0.3	−43	−1.82	0.082

^1^ Abbrev. Std. MW-U statistic = the standardised version of the Mann–Whitney-U test statistic. * Statistically significant *p* < 0.05.

**Table 2:** Comparison of ultra-high field MRI region of interest volumes (cm^3^) in seven affected and three control sheep after adjustment for cerebellum volumes (ROI/cerebellar ROI).

Region	Median of Adjusted Volumes (Control)	Median of Adjusted Volumes (CLN6)	Median Difference	% Reduction	Std. MW-U Statistic ^1^	*p* Value
Corpus callosum	0.14	0.04	0.10	71	2.39	0.017 *
Thalamus	0.12	0.03	0.09	75	2.17	0.033 *
Occipital cortex	0.07	0.02	0.05	71	2.39	0.017 *
Hippocampus	0.056	0.036	0.02	36	1.94	0.067
Striatum	0.20	0.13	0.07	35	2.32	0.024 *
Left side	2.96	2.14	0.82	28	2.39	0.017 *
Arbour vitae	0.44	0.39	0.05	11	0.80	0.517
Cerebral aqueduct	0.004	0.002	0.002	49	0.80	0.517
Cerebellum	1.0	1.0	0.0	0	-	-
Lateral ventricle	0.06	0.08	−0.02	−33	−1.48	0.183

^1^ Abbrev. Std. MW-U statistic = the standardised version of the Mann–Whitney-U test statistic. * Asterisks indicate statistical significance (*p* < 0.05).

## Data Availability

Access to data is available on request.

## Abbreviations

The following abbreviations are used in this manuscript:

*CLN*	Ceroid lipofuscinosis, neuronal (gene)
CLN	Ceroid lipofuscinosis, neuronal (disease or protein)
3D	Three dimensional
MRI	Magnetic resonance imaging
NCL	Neuronal ceroid lipofuscinosis
